# Nanomolar-potency ‘co-potentiator’ therapy for cystic fibrosis caused by a defined subset of minimal function CFTR mutants

**DOI:** 10.1038/s41598-019-54158-2

**Published:** 2019-11-27

**Authors:** Puay-Wah Phuan, Joseph-Anthony Tan, Amber A. Rivera, Lorna Zlock, Dennis W. Nielson, Walter E. Finkbeiner, Peter M. Haggie, Alan S. Verkman

**Affiliations:** 1 Department of Medicine, University of California, San Francisco, San Francisco, CA USA; 2 Department of Pathology, University of California, San Francisco, San Francisco, CA USA; 3 Department of Pediatrics, University of California, San Francisco, San Francisco, CA USA; 4 Department of Physiology, University of California, San Francisco, San Francisco, CA USA

**Keywords:** Drug discovery, Chloride channels

## Abstract

Available CFTR modulators provide no therapeutic benefit for cystic fibrosis (CF) caused by many loss-of-function mutations in the cystic fibrosis transmembrane conductance regulator (CFTR) chloride channel, including N1303K. We previously introduced the concept of ‘co-potentiators’ (combination-potentiators) to rescue CFTR function in some minimal function CFTR mutants. Herein, a screen of ~120,000 drug-like synthetic small molecules identified active co-potentiators of pyrazoloquinoline, piperidine-pyridoindole, tetrahydroquinoline and phenylazepine classes, with EC_50_ down to ~300 nM following initial structure-activity studies. Increased CFTR chloride conductance by up to 8-fold was observed when a co-potentiator (termed ‘Class II potentiator’) was used with a classical potentiator (‘Class I potentiator’) such as VX-770 or GLPG1837. To investigate the range of CFTR mutations benefitted by co-potentiators, 14 CF-associated CFTR mutations were studied in transfected cell models. Co-potentiator efficacy was found for CFTR missense, deletion and nonsense mutations in nucleotide binding domain-2 (NBD2), including W1282X, N1303K, c.3700A > G and Q1313X (with corrector for some mutations). In contrast, CFTR mutations G85E, R334W, R347P, V520F, R560T, A561E, M1101K and R1162X showed no co-potentiator activity, even with corrector. Co-potentiator efficacy was confirmed in primary human bronchial epithelial cell cultures generated from a N1303K homozygous CF subject. The Class II potentiators identified here may have clinical benefit for CF caused by mutations in the NBD2 domain of CFTR.

## Introduction

Cystic fibrosis (CF) is caused by loss of function mutations in the cystic fibrosis transmembrane conductance regulator (CFTR) protein, a cAMP-activated chloride channel^1^. More than 2000 CF-causing CFTR variants have been identified (http://genet.sickkids.on.ca/Home.html). CFTR modulators have been developed that rescue defective cellular processing and cell-surface targeting of mutant CFTRs (correctors) and defective channel gating (potentiators) to restore CFTR anion transport^1–3^. The potentiator Kalydeco (ivacaftor/VX-770) has been approved for CF subjects with gating mutations, including G551D-CFTR and now 38 additional mutations^2^. The corrector/potentiator combinations Orkambi (VX-770 plus lumacaftor/VX-809) and Symdeko (VX-770 plus tezacaftor/VX-661) have been approved for CF subjects that are homozygous for the most common CF-causing CFTR mutation, F508del, or who have one F508del allele and a residual function CFTR mutation^2^. Trikafta, a triple drug combination consisting of two correctors and one potentiator, has recently been approved for CF subjects with one or two F508del alleles^2,4–6^. Trikafta and future CFTR modulators may benefit up to 90% of CF subjects^2^.

Therapeutic approaches are needed for CFTR mutations that are unlikely to benefit from existing modulators – the so-called ‘remaining 10%’^2,7,8^. Non-responsive minimal function CFTR mutations are distributed throughout the CFTR protein and are associated with low CFTR function due to defective channel processing, cell-surface trafficking, and/or channel gating^9^. One such CFTR mutation is N1303K, a missense point mutation located in nucleotide binding domain 2 (NBD2), which is the 5^th^ most common CFTR mutation worldwide accounting for ~2.5% of CFTR mutations (www.cftr2.org). Other minimal function missense CFTR mutants are found in membrane spanning domain (MSD) 1, including G85E and R334W, and MSD2, including L1077P and M1101K. These four CFTR mutants are found in ~1.4% of ~88,000 CF subjects in the CFTR2 database. Premature termination codon (PTC) mutations also have no available therapy, including G542X located in NBD1 and W1282X located in NBD2, which are the 2^nd^ and 4^th^ most common CFTR mutations (5% and 4% allele frequency in CFTR2 database, respectively).

We previously reported that VX-770, when used in combination with a second potentiator identified by high-throughput screening, increased chloride channel function of N1303K-CFTR and the truncated W1282X-CFTR protein product by ~8-fold compared with VX-770 alone^1,10^. This combination-potentiator (co-potentiator) approach was effective as well in increasing the chloride channel function of G551D-CFTR, with ~50% improvement compared with VX-770 alone^11^. Building on these initial results, here we explored the potential utility of co-potentiators for additional minimal function CFTR mutants and identified, by high-throughput screening, novel co-potentiator scaffolds with nanomolar potency. In addition, we provide evidence to support a new classification system (Class I vs. Class II) for potentiators, which predicts synergy in CFTR activation.

## Results

### CFTR mutational space specificity of co-potentiator ASP-11

**Figure 1:**
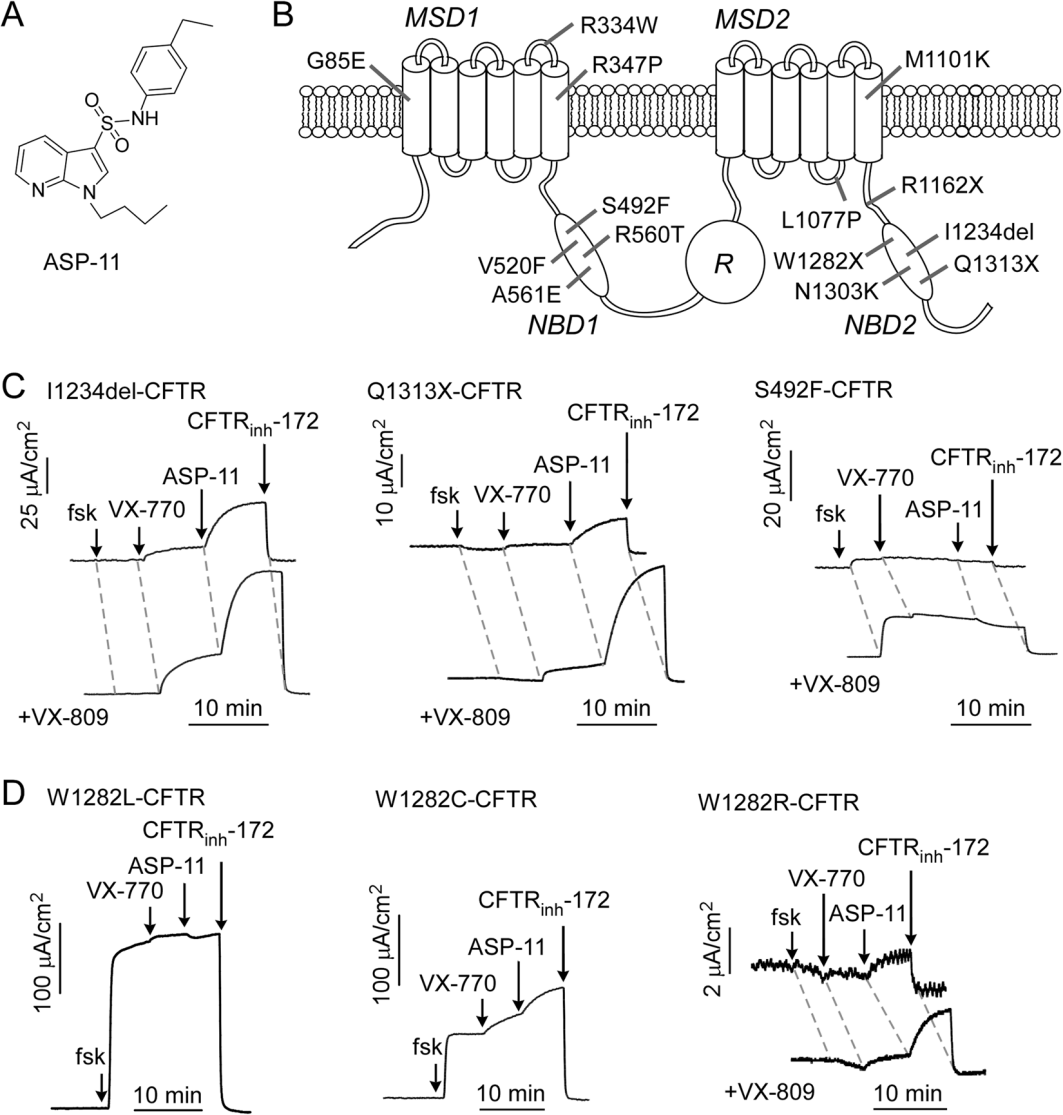
Co-potentiator ASP-11 activity in minimal function CFTR mutants in transfected FRT cells. (**A**) Structure of ASP-11. (**B**) Location of minimal function CFTR mutants studied herein. (**C**) Representative short-circuit current data in FRT cells expressing I1234del-CFTR, Q1313X-CFTR and S492F-CFTR in response to 20 µM forskolin (fsk), 5 µM VX-770, 20 µM ASP-11 and 10 μM CFTR_inh_-172. As indicated, cells were treated with 3 µM VX-809 for 18–24 h prior to measurements. (n = 3) (**D**). Representative short-circuit current data in FRT cells expressing predicted W1282X-CFTR read-through products W1282L-CFTR, W1282R-CFTR and W1282C-CFTR (without and with VX-809 pretreatment) (n = 3). MSD, membrane spanning domain; NBD, nucleotide binding domain; R, regulatory domain.

The arylsulfonamide-pyrrolopyridine ASP-11 (Fig. 1A) was found previously to increase chloride current by ~8-fold over that produced by a high concentration of VX-770 in transfected FRT cells expressing N1303K-CFTR or CFTR_1281_ (the truncation product generated by W1282X-CFTR)^10,11^. To investigate the CFTR mutational space specificity for ASP-11, 12 additional minimal function CFTR mutants were studied (Fig. 1B and Suppl. Table 1). The nine missense mutants studied are located throughout the CFTR protein and included mutations in MSD1 (G85E, R334W, R347P), NBD1 (S492F, V520F, R560T, A561E) and MSD2 (L1077P, M1101K). G85E, V520F and R560T are class II mutations, similar to F508del-CFTR^9^. Two C-terminal PTC mutations, R1162X, and Q1313X, were studied based on the prior W1282X-CFTR data showing benefit of co-potentiators^10^. Lastly, the complex CFTR mutant c.3700A > G was also tested, which introduces a point mutation (I1234V-CFTR) that retains CFTR activity, or a cryptic splice site resulting in a 6-amino acid deletion in NBD2, p.Ile1234_Arg1239del (I1234del-CFTR)^12^. Testing of these NBD2 truncation and deletion mutations was motivated by biochemical and electrophysiological evidence suggesting that incomplete NBD2, including CFTR polypeptides as short as 1217 amino acids, may sometimes allow partial CFTR cell-surface expression and function^13,14^.

**Figure 2:**
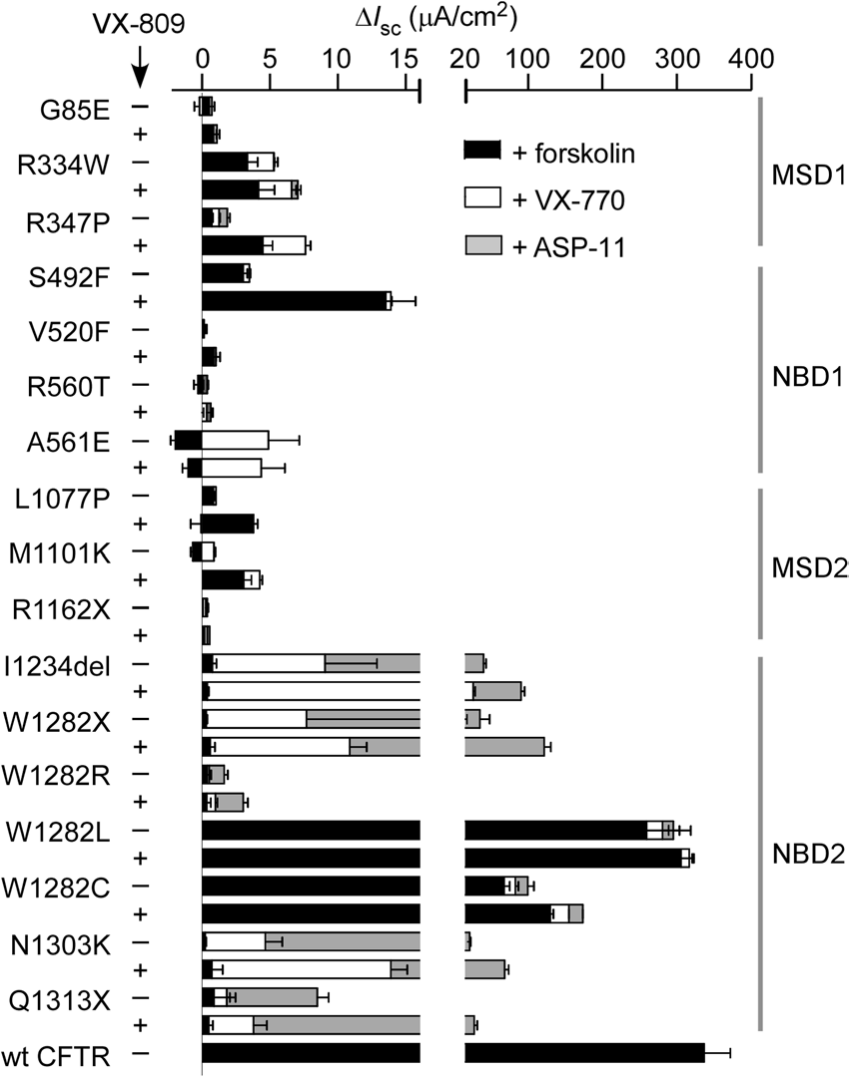
Summary of short-circuit current responses to modulators for CFTR mutants in transfected FRT cells. Responses to indicated CFTR mutants with 20 μM forskolin, 5 µM VX-770, 20 µM ASP-11 (with or without 3 µM VX-809 overnight). For reference, data shown for cells expressing wild type CFTR. Mean ± S.E.M., n = 3.

As shown in Fig. 1C and summarized in Fig. 2, ASP-11 acted in synergy with VX-770 to increase chloride current in FRT cells expressing the NBD2 mutations I1234del- and Q1313X-CFTR by ~3–9-fold compared to VX-770. I1234del- and Q1313X-CFTR-expressing cells were also treated with VX-809 for 24 hours (to consider the effect of available CFTR therapeutics) with 2–3-fold greater currents observed. In contrast, the NBD1 mutation S492F-CFTR showed limited forskolin-stimulated current and no response to VX-770 or ASP-11, though VX-809 increased the forskolin response. Qualitatively similar absence of ASP-11 effect was found for other CFTR mutations located in MSD1 (R334W), NBD1 (V520F and R560T), and MSD2 (L1077P and M1101K), although many of the mutants (R334W, L1077P, M1101K) showed some forskolin activation and VX-770 effect (Fig. 2). In contrast to Q1313X-CFTR (Fig. 2), the C-terminus PTC mutant R1162X-CFTR (predicted to truncate CFTR just after MSD2) did not respond to forskolin, VX-770, ASP-11 or VX-809.

Compounds that promote read-through of PTCs to generate full-length CFTR have shown limited efficacy in cell culture studies^15–17^. Though the read-through drug ataluren (PTC124) was ineffective in clinical trials^18^, newer compounds are in development. We investigated the ability of ASP-11 to activate chloride current in predicted W1282X-CFTR read-through products. G418 action on W1282X was found to insert mainly leucine or cysteine at position 1282^19^, and ataluren to insert arginine^17^. FRT cell lines were generated that stably expressing W1282L-, W1282C- and W1282R-CFTR. W1282L-CFTR showed robust forskolin stimulation with little additional VX-770 or ASP-11 effect (Figs. 1D and 2). W1282C-CFTR showed less forskolin response but significant further effects of VX-770 and ASP-11, and W1282R-CFTR showed minimal function (Figs. 1D and 2). Together, the data as summarized in Fig. 2 suggest that several missense, deletion and truncation mutations in NBD2 may be amenable to co-potentiator action.

### Classification of potentiators to predict synergy

**Figure 3:**
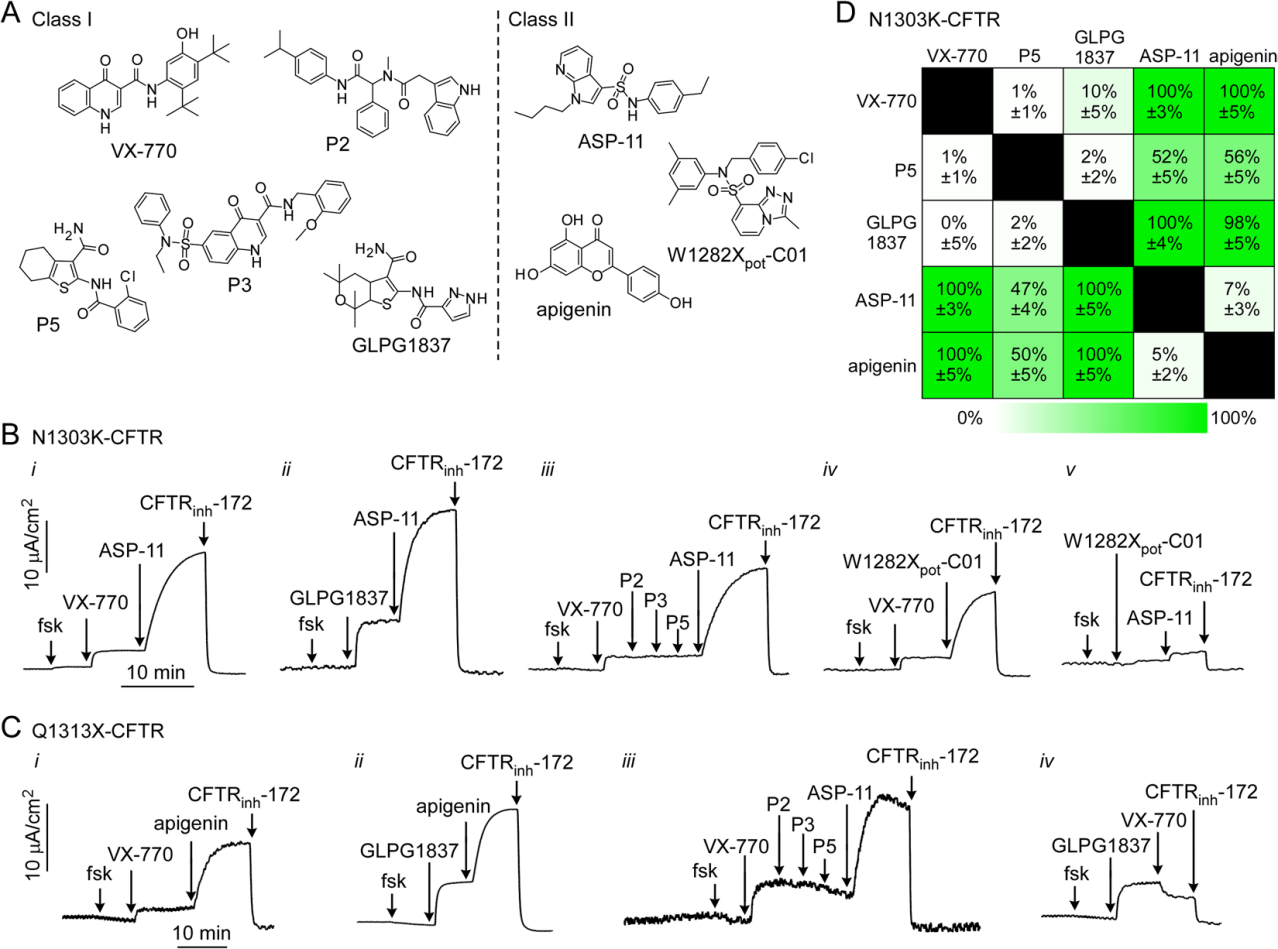
Definition of Class I and II CFTR potentiators. (**A**) Chemical structures of Class I and II potentiators. (**B**) Short-circuit current in FRT cells expressing N1303K-CFTR in response to forskolin and indicated potentiators. Concentrations: 20 μM forskolin, 5 μM VX-770, 20 μM ASP-11, 20 μM GLPG1837, 20 μM P2, 20 μM P3, 20 μM P5, 20 μM W1282X_pot_-C01, and 10 μM CFTR_inh_-172. **C**. Short-circuit current in FRT cells expressing Q1313X-CFTR in response to forskolin and indicated potentiators. Concentrations: as in panel B, and 25 μM apigenin. (**D**) Summary of relative N1303K-CFTR activation in response to combinations of Class I and Class II potentiators. Mean ± S.E.M., n = 3.

Based on prior studies^10,11,20^, we hypothesized that distinct binding sites may be required to bind a potentiator and co-potentiator to rescue channel activity of CFTR mutants. We defined the potentiator VX-770 as a Class I compound, and co-potentiator ASP-11 as a Class II compound (Fig. 3A,D). Short-circuit current measurements in N1303K-CFTR-expressing FRT cells confirmed synergy when ASP-11 was added after VX-770 (Fig. 3Bi). Similar results were found for ASP-11 added after GLPG1837 (Fig. 3Bii), suggesting that GLPG1837 is a Class I potentiator. Additions of P2, P3 and P5 (potentiator activity confirmed in separate studies, not shown) did not further increase current when added after VX-770 (Fig. 3Biii), indicating that these compounds also belong to Class I. Interestingly, VX-770 addition after GLPG1837 mildly reduced current (data not shown), consistent with competitive binding between VX-770 and GLPG1837 as reported for G551D-CFTR^20^. Another co-potentiator previously reported to activate W1282X-CFTR, W1282X_pot_-C01^10^, activated N1303K-CFTR when used with VX-770 (Fig. 3Biv), demonstrating this compound is a Class II potentiator. Finally, two Class II potentiators, W1282X_pot_-C01 and ASP-11, produced little activation when added together (Fig. 3Bv). In each experiment, CFTR_inh_-172 confirmed that current was from CFTR.

Studies on FRT cells expressing Q1313X-CFTR produced similar data (Fig. 3C). Addition of the Class I potentiators VX-770 (Fig. 3Ci) or GLPG1837 (Fig. 3cii) followed by the Class II potentiator apigenin showed synergy. In contrast, sequential additions of Class I potentiators P2, P3 and P5 did not elevate current following VX-770, whereas Class II potentiator ASP-11 did (Fig. 3ciii). As seen for N1303K-CFTR and G551D-CFTR^20^, VX-770 added after GLPG1837 reduced current in Q1313X-CFTR expressing cells (Fig. 3civ). As summarized in Fig. 3D for N1303K-CFTR, synergy was seen for Class I and Class II compounds used together, but not for combinations of Class I-Class I or Class II-Class II compounds. Similar results were seen for Q1313X-CFTR (Suppl. Figs. 1 and 2).

### Novel co-potentiator scaffolds identified by high-throughput screening

**Figure 4:**
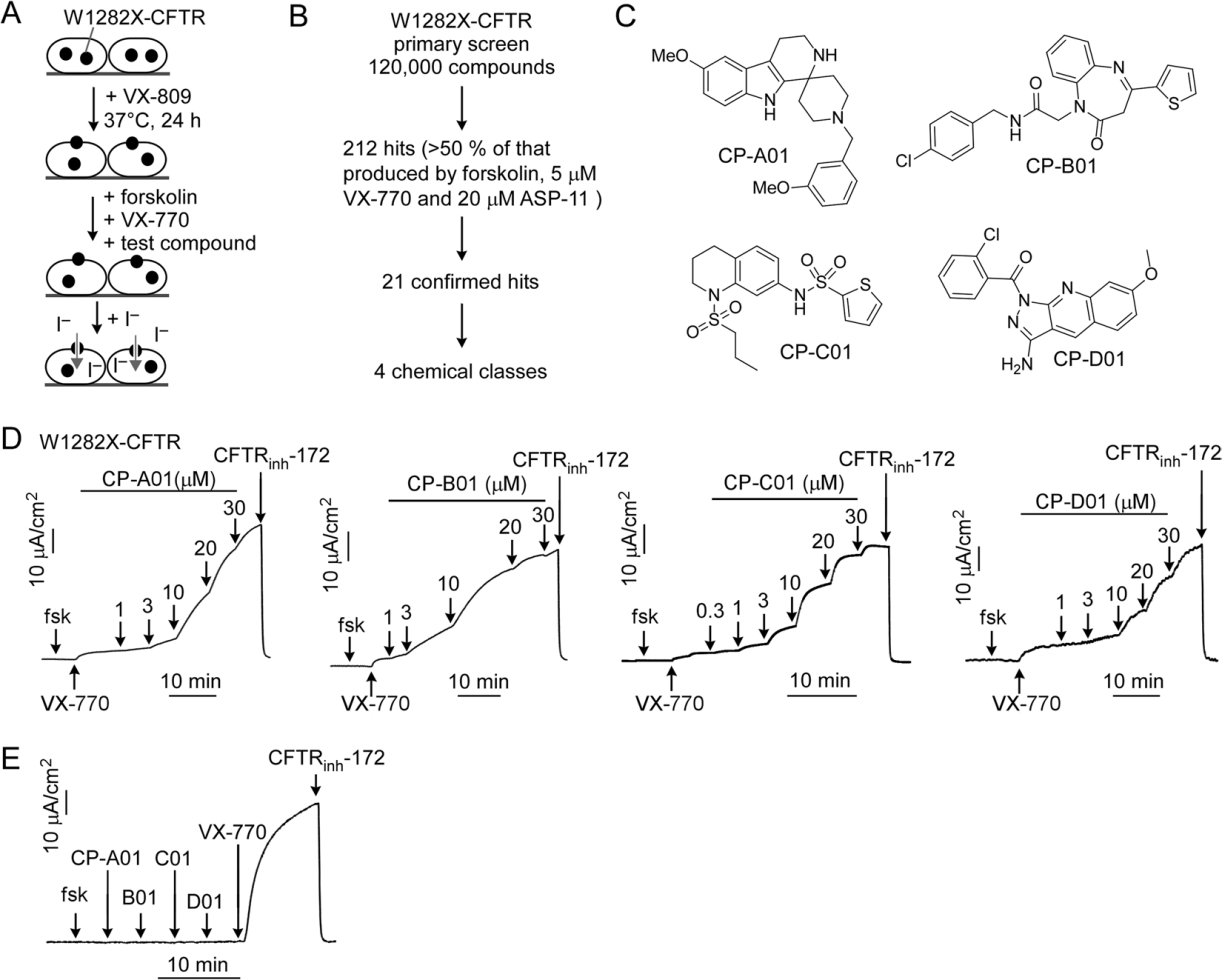
Novel co-potentiators identified by high-throughput screening. (**A)** Assay design. (**B**) Summary of screening workflow and results. (**C**) Chemical structures of novel co-potentiators identified by screening. (**D**) Representative short-circuit current data in FRT cells expressing W1282X-CFTR. Concentrations: 20 μM forskolin (fsk), 5 μM VX-770 (n = 3). (**E**) Effects of sequential addition of 20 μM forskolin followed by 20 μM of A-01, B-01, C-01 and D-01, and 5 μM of VX-770 in FRT cells expressing W1282X-CFTR. CFTR_inh_-172 was used at 10 μM in all experiments.

Given the utility of co-potentiators as possible CF therapeutics for several minimal function CFTR mutants, a screen was done to identify novel co-potentiator scaffolds. Screening used FRT cells stably expressing W1282X-CFTR and the halide-sensitive EYFP-H148Q/I152L/F46L (YFP) that were treated for 24 hours with 3 μM VX-809 to increase CFTR_1281_ cell surface expression (Fig. 4A). Just prior to assay cells were treated for 10 min with test compounds at 25 μM together with 20 μM forskolin and 15 nM VX-770. CFTR channel activity was deduced from the initial rate of YFP fluorescence quenching in response to addition of iodide-substituted phosphate buffered saline. Primary screening of 120,000 drug-like synthetic small molecules identified 212 compounds giving channel activity >50% of that produced by forskolin, VX-770 and 20 μM ASP-11 (Fig. 4B). After initial confirmation with plate reader assays, short-circuit current measurement revealed the 21 most active compounds of four chemical classes, which were further studied. The most active compounds included piperidinepyridoindole CP-A01, phenylazepine CP-B01, tetrahydroquinoline CP-C01 and pyrazoloquinoline CP-D01 (Fig. 4C). Concentration-dependence measurements in VX-809-corrected W1282X-CFTR-expressing FRT cells in the presence of forskolin and 5 μM VX-770 demonstrated an ~6-fold increase in VX-770 current with EC_50_ of 10 μM, 5 μM, 8 μM and 15 μM for CP- A01, B-01, C01 and D01, respectively (Fig. 4D). Each of these compounds added to VX-809-corrected FRT cells expressing W1282X-CFTR produced little current without VX-770 (Fig. 4E). As found for ASP-11^9,10^, synergy of W1282X-CFTR activation by VX-770 and the new Class II potentiators did not depend on the order of compound addition (data not shown).

### Structure-activity relationship studies of piperidinepyridoindole and pyrazoloquinoline co-potentiators

**Figure 5:**
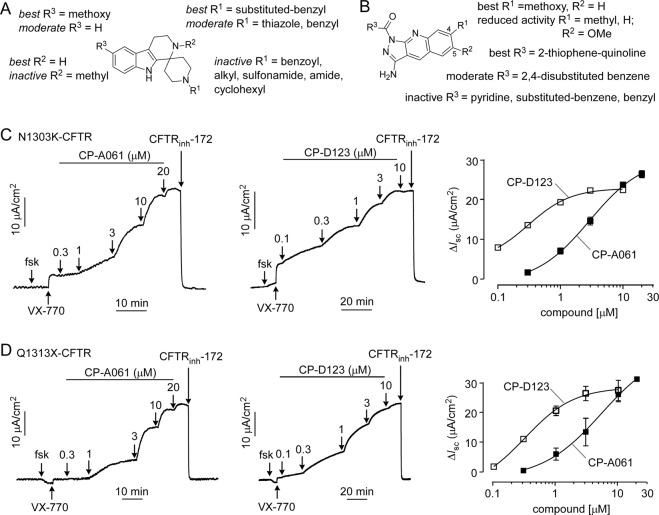
Structure-activity analysis of piperidine-pyridoindole and pyrazoloquinoline co-potentiators. (**A**) Structural determinants of piperidine-pyridoindole (CP-Axxx) and (**B**) pyrazoloquinoline (CP-Dxxx) co-potentiator activity. (**C**) Short-circuit current in FRT cells expressing N1303K-CFTR showing responses to 20 µM forskolin (fsk), 5 µM VX-770, and indicated concentrations of CP-A061 (*left*) and CP-D123 (*center)*. (*right*) Summary of concentration-dependence data. (**D**) Short-circuit current in FRT cells expressing Q1313X-CFTR done as in panel C. (*right*) Summary of concentration-dependence data.

To establish structure-activity relationships, 240 commercially available piperidinepyridoindole analogs and 160 pyrazoloquinoline analogs were tested in FRT cells expressing W1282X-CFTR. Figure 5A summarizes the structural determinants for activity for the piperidinepyridoindoles reported in Table 1 (CP-Axx designations). In general, the methoxy substituent on the 4-position (R^3^) on the pyridoindole ring increased potency (compare A01 vs A534). N-methylation on the pyridoindole ring abolished activity (compare A534 vs A600). For substituent R^1^ on the piperidine ring, substituted benzyl gave greatest activity (A061 and A662). Other R^1^ substituents, including sulfonamide (A145), alkyl (A764) and carbocyclic (A714), reduced activity. Changing the benzylic carbon from methylene (CH_2_) to ketone (C=O) abolished activity (A815, A350 and A956). A061 with R^1^ being 2,4-difluoro-benzyl was the most potent analog.

**Table 1:** Structure-activity data of selected piperidine-pyridoindole analogs.


Compound	R^1^	R^2^	R^3^	EC_50_ (μM)	Relative V_max_ (%)*
A01	3-methoxy-benzyl	H	OMe	10	100
A061	2,4-difluoro-benzyl	H	OMe	2.0	100
A662	3,4-difluoro-benzyl	H	OMe	2.6	100
A666		H	OMe	5.1	91
A357	benzyl	H	H	16	31
A534	3-methoxy-benzyl	H	H	13	24
A600	3-methoxy-benzyl	Me	H	>30	4
A714	cyclohexyl	H	OMe	>30	18
A815		H	H	>30	-4
A350		H	OMe	>30	7
A956		H	OMe	>30	6
A145	SO_2_-Me	H	OMe	>30	7
A764	Me	H	OMe	>30	6

*100% V_max_ corresponds to 20 μM ASP-11.

Figure 5B summarizes the structural determinants for the pyrazoloquinolines reported in Table 2 (CP-Dxx designations). The position of the methoxy on the quinoline ring affected activity as changing from the 4^th^ to 5^th^ position greatly reduced potency (D038 vs D138). Replacing the electron-donating methoxy group to electron-neutral methyl group also reduced activity (D010 vs D136). For R^3^, pyridine (D003), benzyl (D035) and substituted methyl-pyrazole (D086) abolished activity. Substituted benzenes had a range of potencies with 2,4-disubstituted compounds including 2-chloro-4-fluorobenzene (D018) and 2-chloro-4-nitrobenzene (D038) being the most potent. The D123 pyrazoloquinoline with R^3^ substituted with thiophene-quinoline heterocycle gave the best potency.

**Table 2:** Structure-activity data of selected pyrazoloquinoline analogs.


Compound	R^1^	R^2^	R^3^	EC_50_ (μM)	Relative V_max_ (%)*
D001	OMe	H	2-chlorobenzene	15	90
D003	OMe	H	4-pyridine	>30	1
D010	OMe	H	4-fluorobenzene	3.6	24
D012	OMe	H	3,4,5-trimethoxybenzene	>30	3
D018	OMe	H	2-chloro-4-fluoro-benzene	2.4	92
D025	OMe	H	3-methyl-4-nitro-benzene	>30	3
D035	OMe	H		>30	1
D036	OMe	H	3-nitro-4-chloro-benzene	>30	11
D038	OMe	H	2-chloro-4-nitro-benzene	1.7	100
D086	OMe	H		>30	3
D123	OMe	H		0.3	100
D136	Me	H	4-fluorobenzene	18	11
D138	H	OMe	2-chloro-4-nitro-benzene	3.4	21

*100% V_max_ corresponds to 20 μM ASP-11.

Short-circuit current measurements were done for the most potent piperidinepyridoindole (CP-A061) and pyrazoloquinoline (CP-D123). Figures 5C,D show data in N1303K- and Q1313X-expressing FRT cells. Following forskolin and VX-770, concentration-dependent increases in current were seen following addition of co-potentiators, with current fully inhibited by CFTR_inh_-172. The calculated EC_50_ values were 2.9 μM and 300 nM for CP-A061 and CP-D123, respectively, in the FRT cells expressing N1303K-CFTR. Similar EC_50_ values of 5 μM and 320 nM for CP-A061 and CP-D123 were found in FRT cells expressing Q1313X-CFTR.

### Co-potentiator activity in human CF airway epithelial cell models and primary cell cultures

**Figure 6:**
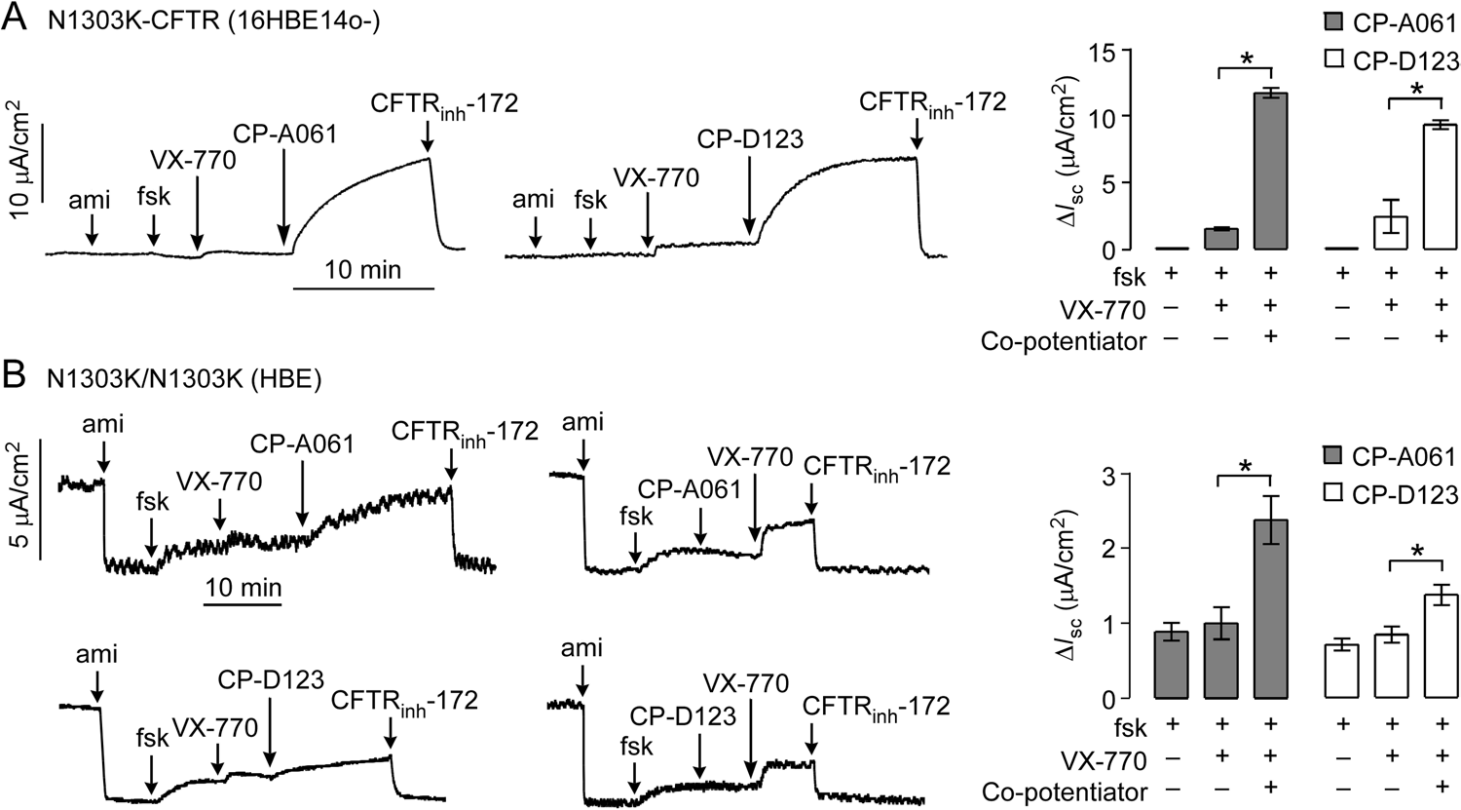
N1303K-CFTR activity in human airway epithelial cell cultures. (**A**) Short-circuit current in gene-edited 16HBE14o- cells expressing N1303K-CFTR. Concentrations: 20 µM amiloride, 20 µM forskolin, 5 µM VX-770, 20 µM CP-A061 and CP-D123, and 10 µM CFTR_inh_-172. Representative original curves (*left and center*) and summary of changes in current (∆Isc) (*right*). Mean ± S.E.M., n = 3, *P < 0.05). (**B**) Short-circuit current in primary cultures of human bronchial epithelial cells from a homozygous N1303K-CFTR CF subject. Concentrations were as in panel A. Representative original curves (*left and center*) and summary of changes in current (∆Isc) (*right*). Mean ± S.E.M., n = 3, *P < 0.05.

To test the efficacy of new co-potentiators in human airway cell models, short-circuit current was measured in 16HBE14o- human airway epithelial cells in which the endogenous CFTR gene was edited to contain the N1303K mutation (16HBE-N1303Kge^21^) and in primary cultures of human bronchial epithelial cells from a N1303K homozygous CF subject. Addition of forskolin and then VX-770 to 16HBE-N1303Kge cells gave a limited response (Fig. 6A). Subsequent addition of CP-A061 or CP-D123 produced CFTR_inh_-172-inhibitable responses of ~10 μA/cm^2^, ~6-fold greater that that produced by VX-770 alone. Increased short-circuit current was also found for these co-potentiators in the primary human bronchial epithelial cell cultures (Fig. 6B).

## Discussion

This purpose of this study was to develop a therapeutic strategy for CF subjects with CFTR mutations that do not respond significantly to the available drugs Kalydeco, Orkambi and Symdeko. This work extended our prior data on CFTR modulators, called co-potentiators, that act in synergy with VX-770 to increase channel activity of W1282X-CFTR and N1303K-CFTR by ~8-fold, and of G551D-CFTR by ~1.5-fold, compared to VX-770 alone^10,11^. Here, high-throughput screening and preliminary structure-activity studies identified novel co-potentiator scaffolds with drug-like properties with EC_50_ down to 300 nM, which acted in synergy with VX-770 and other Class I potentiators to increase CFTR chloride current. An interesting observation was that the potentiator/co-potentiator paradigm was effective on a variety of missense, nonsense and deletion mutations specifically in NBD2 of CFTR. Whether analogous combination potentiators can be developed for residual or minimal function mutants in other regions of the CFTR protein, perhaps with next-generation corrector(s), is not known but warrants further consideration.

VX-770 is currently approved for CF subjects with one copy of one of 38 mutations located throughout the CFTR sequence, including MSD1 (e.g. D110H, E193K), MSD2 (A1067T, R1070W), NBD1 (G551D, D579G), NBD2 (G1244E, G1349D), and other regions (E56K, P67L in the lasso domain)^2,22,23^. Based on *in silico* docking and mutagenesis studies, two potential binding sites were identified for the Class I compounds VX-770 and GLPG1837^24^. The putative binding sites are located at the interface of the CFTR transmembrane domains involving residues D924, N1138 and S1141, or residues F229, F236, Y304, F312 and F931^24^. Recently, cryo-electron microscopy confirmed that VX-770 and GLPG1837 bind at the same site within the protein-lipid interface in a pocket formed by transmembrane helices 4, 5 and 8^25^. Evaluation of potential binding sites by alanine substitution revealed that a network of residues (including F236, Y304, F312 and F931, as well as L233, F305 and S308) interact directly with both VX-770 and GLPG1837^25^. Using a pharmacological approach, we found evidence that several previously reported potentiators including P2, P3 and P5 also bind at or near the VX-770 and GLPG1837 binding site. Given the broad efficacy of VX-770 for mutations throughout the CFTR protein, and the absence of large differences in CFTR structure with vs. without bound potentiator, it is likely that Class I potentiators thermodynamically alter the closed-open equilibrium of CFTR^25^. The Class II potentiators identified here act in synergy with VX-770 and other Class I compounds to rescue the defective gating of CFTR mutations in NBD2. At present, the putative binding site(s) of Class II potentiators is unknown, though early *in silico* docking studies^26^ showed that genistein, which is structurally similar to the Class II potentiator apigenin, binds at the NBD dimer interface. We therefore speculate that the Class II potentiators may bind to CFTR in a manner that stabilizes partial or misfolded NBD2 structurally or thermodynamically. Because potentiators may produce both long-range and local conformational changes, as well as thermodynamic alterations to increase conductance of mutant CFTRs, potentiator synergy screening as done here may be useful for selected mutations in other regions of the CFTR protein.

The results here suggest that a variety of missense, nonsense and deletion mutations in NBD2, including N1303K- and I1234del-CFTR, can be benefitted by two distinct potentiators. In prior studies on the responses of >50 rare CFTR missense mutations to VX-770 and VX-809, N1303K-CFTR was not responsive to VX-770 and showed very limited response to VX-809^27^. This is consistent with the notion that the N1303K mutation causes defective CFTR folding, regulation and gating^9^. Biochemical analysis of N1303K-CFTR processing in various cell systems, including FRT cells^28^ and 16HBE14o- cells^21^, showed little complex glycosylated CFTR (band C) with or without VX-809 (Hillary Valley and Martin Mense, personal communication). Previous studies on the relationship of CFTR glycosylation to function suggest that the addition of carbohydrate to CFTR is not a necessary prerequisite for CFTR targeting to the plasma membrane or functioning as a cAMP-stimulated chloride channel^29,30^. Our data showing significant current from three N1303K-CFTR expressing cell models suggest that some core glycosylated N1303K-CFTR is present on the plasma membrane. Han *et al*^27^. reported diverse responses to CFTR modulators – some mutations (P5L, G27R, S492F, Y1032C) responded to VX-809 but not VX-770, some (M348V) to VX-770 but not VX-809, and some (G85E, R560T, A561E, Y563N) with no response. We found robust activation of N1303K-CFTR with co-potentiators in the absence of a corrector in a human airway epithelial cell line expressing endogenous levels of gene-edited CFTR (Fig. 6A). In primary human bronchial cell cultures from a homozygous N1303K-CFTR subject, CP-A061 produced ~2.5 μA/cm^2^ of CFTR_inh_-172-inhibitable current (Fig. 6B). Based on measurements done using similarly cultured non-CF and CF human airway epithelial cell cultures, we estimate that 2.5 μA/cm^2^ of CFTR current is equivalent to ~20% of wildtype CFTR activity, which is potentially of clinical benefit. This value would further increase with use of an effective corrector to increase N1303K-CFTR cell-surface expression.

It is hard to estimate the number CF subjects that might benefit from co-potentiator therapy. The N1303K allele is found in 2,147 subjects in the CFTR2 databases, of which 99 are homozygous and >400 would not be benefitted by VX-770 or therapies that targeting one F508del-CFTR allele. Similarly, c.3700A > G is found in 28 subjects, of which 5 are homozygous. It is noted that many countries in which N1303K and c.3700A > G are prevalent do not contribute to the CFTR2 database^31,32^. We previously showed that ASP-11 activates G551D-CFTR, as do the new co-potentiators identified here (not shown). The G551D allele is found in ~3000 CF subjects in CFTR2, including 69 homozygous subjects. Although approved to treat 38 CF variants with residual functions, Ivacaftor (VX-770) however does not fully restore CFTR function of some of these CF variants with gating mutations (e.g. G551D-CFTR)^33^ to wild-type level. Co-potentiator therapy might thus be used to further increase CFTR function, with greater health benefits for CF patients with these gating mutations. In addition, the co-potentiators were effective in increasing CFTR chloride current for several truncated forms of CFTR resulting from premature termination codons (PTCs) located in NBD2. PTCs result in nonsense-mediated degradation (NMD) of transcript resulting in reduced synthesis of truncated protein products^34,35^. CFTR transcript levels have been reported from ~10–75% of levels in non-CF cells^36,37^, though one study reported complete absence of W1282X-CFTR transcript in cells from a single CF subject^38^. Co-potentiators may thus be therapeutically beneficial for PTCs in NBD2, alone if sufficient transcript is present, or in combination with other drugs such as NMD inhibitors or read-through agents.

Limited information has been reported on biological activities for the pyrazoloquinoline and piperidine-pyridoindole classes of compounds. Some acylated pyrazoloquinolines showed *in vitro* antibacterial activity by inhibition of bacterial serine/threonine protein kinases^39^. Pyrazoloquiolines from a chemical library screen were also identified as enhancers of siRNA delivery^40^. Piperidine-pyridoindoles have been reported as ligands of 5-HT_1A_ receptors^41^, and their amino-alkylated analogs showed anxiolytic activity in mice^42^. Synthetically, both pyrazoloquinoline and piperidine-pyridoindole scaffolds can be prepared in four-to-seven steps in a convergent manner from commercially available starting chemicals to allow facile and rapid diversification of these scaffolds. CP-A061 and CP-D123 have drug-like properties, including the presence of multiple hydrogen bond acceptors, as well as aLogP, molecular weight, and topological polar surface areas of 4.39, 397 and 40.3 Å^2^, respectively, for CP-A061, and 4.97, 451 and 95.9 Å^2^, respectively, for CP-D123.

In summary, the present study identified novel co-potentiator scaffolds with nanomolar potency that, in synergy with Class I potentiators such as VX-770, activated CFTRs with NBD2 mutations including N1303K-CFTR. Potentiator/co-potentiator combination therapy may be effective in a subset of minimal function missense, nonsense and deletion mutations in CFTR that cause cystic fibrosis and are not responsive to current CFTR modulator combinations.

## Materials and Methods

### Chemicals

VX-809, VX-770, GLPG1837 and CFTR_inh_-172 were purchased from Selleck Chemicals (Boston, MA). Potentiators P2 (PG-01^43^), P3 (SF-03^43^) and P5 (∆F508_act_-02^44^) were obtained from an in-house repository of CFTR modulators. For screening, 120,000 diverse drug-like synthetic compounds (ChemDiv Inc., San Diego, CA) were tested. Other chemicals were purchased from Sigma unless otherwise stated.

### Complementary DNA constructs

Complementary DNAs (cDNAs) for the I1234del-, W1282C/L/R and Q1313X- mutants CFTRs were generated using standard techniques. In brief, gBLOCK gene fragment (Integrated DNA Technology, Coralville, IA) were synthesized and introduced into full-length CFTR cDNA in the vector pcDNA3.1/Zeo (+) (Invitrogen). For subcloning, I1234del-CFTR was generated using a *Hin*dIII site at position 3171–3176 of the CFTR cDNA; for W1282C/L/R and Q1313X-CFTR a *Bst*XI site at position 3801–3812 of CFTR cDNA was used. The mutated CFTR cDNAs were subcloned into vector pIRESpuro3 (Clontech, Mountain View, CA) using *Nhe*I and *Not*I restriction sites. All constructs were confirmed by sequencing.

### Cell culture models

Fischer rat thyroid (FRT) cells were cultured in Kaign’s modified Ham’s F-12 medium supplemented with 10% FBS, 2 mM L-glutamine, 100 units/ml penicillin, 100 μg/ml streptomycin, 18 μg/ml myoinositol, and 45 μg/ml ascorbic acid. To generate FRT cells stably expressing I1234del-, W1282C/L/R and Q1313X-CFTR, cells were transfected with pIRESpuro3-based vectors and clonal cell lines were isolated after inclusion of 0.15 µg/ml puromycin (Invitrogen) in cell culture medium. FRT cell lines expressing wild type, W1282X- and N1303K-CFTR were cultured as reported^10,11,45^. FRT cells lines expressing G85E-, R334W-, R347P-, S492F-, V520F-, R560T-, A561E-, L1077P-, M1101K- and R1162X-CFTR were a generous gift from Drs. Andras Rab and Jeong Hong (Emory University) and cultured as described^27^. Gene edited 16HBE14o- cells expressing N1303K-CFTR were provided by the CFFT Lab, and were cultured as described^21^.

### Primary human bronchial and nasal epithelial cell cultures

Human bronchial epithelial cells isolated from a lung transplant from a N1303K homozygous CF subject were provided by Scott H. Randell (Marsico Lung Institute, The University of North Carolina at Chapel Hill, USA). The cells were obtained under protocol #03-1396 approved by the University of North Carolina at Chapel Hill Biomedical Institutional Review Board. Cells were isolated, conditionally reprogrammed, and expanded as described^10,46^.

### High-throughput screening

High-throughput screening was performed using a semi-automated screening platform (Beckman, Fullerton, CA) as described^10^. In short, FRT cells expressing W1282X and halide-sensitive YFP were plated onto 96-well tissue-culture plates at a density of 20,000 cells/well (black-walled, clear-bottom)(Corning). The cells were grown for 24 h at 37 °C to ~90% confluency before treatment with 3 μM VX-809 for additional 24 hours. Cells were then washed twice with PBS. Cells were then added with 100 μl of PBS containing forskolin (10 μM), VX-770 (15 nM) and test compounds (25 μM) and incubated for 10 min prior to assay of CFTR activity. All plates contained wells with positive (5 μM VX-770 + 20 μM ASP-11) and negative (5 μM VX-770) controls. Assays were done using a BMG Labtech FLUOstar OMEGA plate reader (Cary, NC). Each well was read over 12 s with initial fluorescence intensity recorded for 2 s before addition of 100 μl of NaI-substituted PBS (137 mM NaCl replaced with NaI). Initial iodide influx rate was computed from fluorescence intensity using single exponential regression.

### Short-circuit current measurements

Short-circuit current was measured on cells cultured on Snapwell clear permeable supports (Corning) as described^10,11^. Hemichambers were connected to a DVC-1000 voltage clamp (World Precision Instruments Inc., Sarasota, FL) via Ag/AgCl electrodes and 3 M KCl agar bridges for recording of the short-circuit current. For human airway epithelial cells, symmetrical HCO_3_^−^-buffered solutions (in mM: 120 NaCl, 5 KCl, 1 MgCl_2_, 1 CaCl_2_, 5 Hepes, 25 NaHCO_3_, 10 glucose, pH 7.4) for basolateral and apical side were used. For FRT cells, the basolateral membrane was permeabilized with 250 μg/ml amphotericin B, and experiments were done with a chloride gradient using a HCO_3_^−^-buffered solutions (Basolateral (mM): 120 NaCl, 5 KCl, 1 MgCl_2_, 1 CaCl_2_, 5 Hepes, 25 NaHCO_3_, 10 glucose, pH 7.4; apical (mM): 60 NaCl, 60 mM sodium gluconate, 5 KCl, 1 MgCl_2_, 1 CaCl_2_, 5 Hepes, 25 NaHCO_3_, 10 glucose, pH 7.4). All cells were equilibrated with 95% O_2_, 5% CO_2_ and maintained at 37 °C during experiments.

### Data analysis

For statistical analysis, GraphPad Prism software (GraphPad Inc., San Diego, CA, USA) was used. Statistical significance was determined using the Mann-Whitney test, with *P* < 0.05 considered significant. For determination of EC_50_ values, non-linear regression to a single-site inhibition model was used.

## Supplementary information

supplementary info
