# Supplementary material for: Nanomolar-potency ‘co-potentiator’ therapy for cystic fibrosis caused by a defined subset of minimal function CFTR mutants
Source: Sci Rep. 2019 Nov 27;9:17640. doi: 10.1038/s41598-019-54158-2 (PMC6881293; doi:10.1038/s41598-019-54158-2)
Supplement: Supplementary file 1 — supplementary info [file 41598_2019_54158_MOESM1_ESM.pdf]

Supplementary Material

Nanomolar-potency ‘co-potentiator’ therapy for cystic fibrosis caused by a defined subset of minimal function CFTR mutants

Puay-Wah Phuan[1], Joseph-Anthony Tan[1], Amber A. Rivera[1], Lorna Zlock[2], Dennis Nielson[3], Walter E. Finkbeiner[2], Peter M. Haggie[1] and A.S. Verkman[1,4]

[1]Department of Medicine, [2]Department of Pathology, [3]Department of Pediatrics, and [4]Department of Physiology, University of California, San Francisco, San Francisco, CA, USA

Corresponding author: Puay-Wah Phuan. Department of Medicine, University of California, San Francisco, San Francisco, CA, USA. puay-wah.phuan@ucsf.edu

CFTR mutation	Classification of defect
G85E	folding/maturation defect
R334W	folding/maturation and gating/conductance defects
R347P	folding/maturation and gating/conductance defects
S492F	folding/maturation and gating defect
V520F	folding/maturation defect
R560T	folding/maturation defect
A561E	folding/maturation, gating/conductance defects, stability
L1077P	folding/maturation, gating/conductance defects, stability
M1101K	folding/maturation defect
R1162X	stability/truncation
I1234del	deletion
W1282X	stability/truncation
N1303K	folding/maturation, gating/conductance defects
Q1313X	stability/truncation

Supplemental Table 1. Classification of cystic fibrosis mutations studied, as adapted from Veit, G. et al. Mol. Biol. Cell 27, 424-433 (2016).

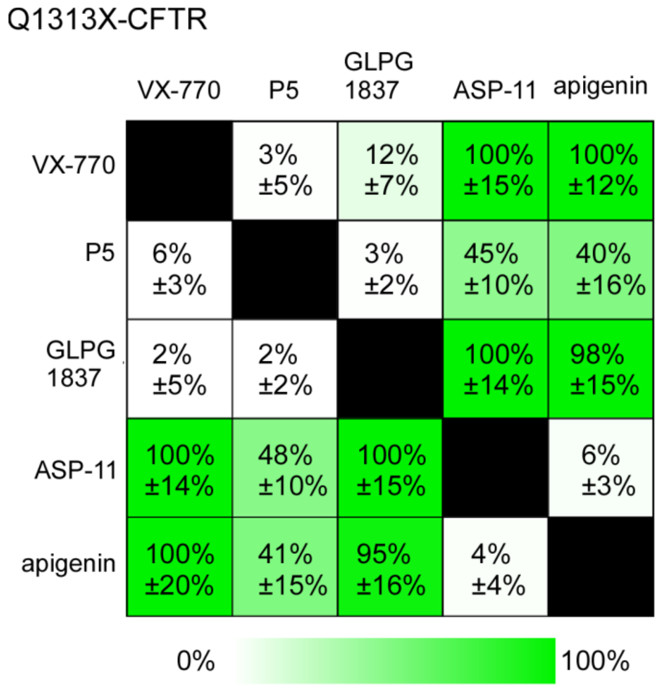

Supplemental Figure 1. Summary of relative Q1313X-CFTR activation in response to combinations of Class I and Class II potentiators. Mean ± S.E.M., n=3.

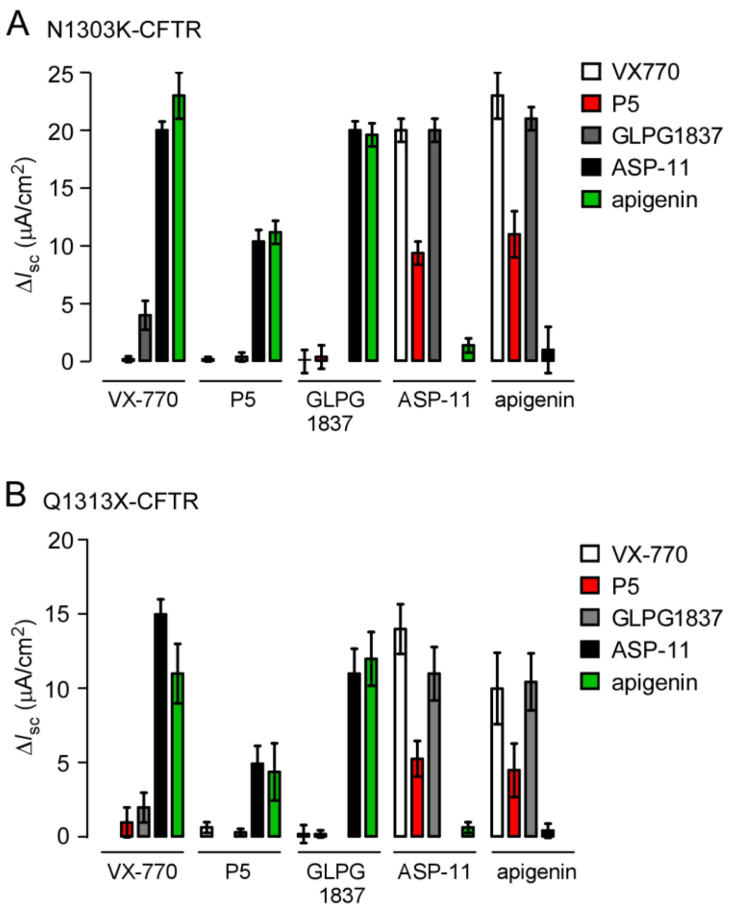

Supplemental Figure 2. Summary of short-circuit current responses to CFTR modulator combinations for **A**) N1303K-CFTR and **B**) Q1313X-CFTR in transfected FRT cells. Concentrations: 5 µM VX-770, 20 µM P5, 20 µM ASP-11, 25 µM apigenin. 20 µM forskolin. Mean ± S.E.M., n=3.
